# Statistical methods in randomised controlled trials for delirium

**DOI:** 10.1016/j.jpsychores.2012.06.002

**Published:** 2012-09

**Authors:** Daniel Farewell, Tayyeb A. Tahir, Jonathan Bisson

**Affiliations:** a Cochrane Institute of Primary Care and Public Health, School of Medicine, Cardiff University, Cardiff, UK; b Department of Liaison Psychiatry, University Hospital of Wales, Cardiff, UK; c School of Medicine, Cardiff University, Cardiff, UK; d Cardiff and Vale University Health Board, University Hospital of Wales, Cardiff, UK

**Keywords:** Delirium, Nonlinear models, Randomised controlled trials, Statistical methods

## Abstract

**Objective:**

The analysis of clinical trials in delirium is typically complicated by treatment dropouts and by the fact that even untreated individuals may have a good prognosis. These features of delirium trials warrant special statistical attention; implications for each stage of a trial planning process are described.

**Methods:**

Choice of outcome, patient sample, and data collection in delirium trials are discussed. Descriptions are given, together with examples, of time-to-event, imputation-based, linear and nonlinear models for the analysis of randomised controlled trials for delirium.

**Results:**

Imputation allows evaluation of the plausibility of individual recovery trajectories, but some simple imputations are found to be unsuitable for delirium research. Time-to-event and nonlinear models encourage a global perspective on analysis, which is often preferable to cross-sectional end-of-trial assessments. It is suggested that nonlinear random effects models for longitudinal trajectories are particularly suitable in a delirium context.

**Conclusion:**

It is hoped that the methods described, and nonlinear models in particular, will play a part in convincing analyses of future delirium research.

## Introduction

The objective of the present paper is to consider some of the statistical issues that arise during the conduct of randomised trials in delirium. Delirium is a serious and complex neuropsychiatric syndrome that can fluctuate and improve irrespective of the treatment given, which raises important statistical questions at each stage of a scientific study. The principal focus herein is analysis and, in particular, on the analytical challenges raised by the common problem of sample attrition. This chosen emphasis is not because analysis is the most important part of a clinical trial, but because it is here that there is greatest potential for improvement over current practice.

Patients may drop out because of adverse reaction to treatment, rapid deterioration of condition, relapse or recovery. Dropouts necessarily lead to a loss of statistical power, but may also raise concerns regarding the validity of conclusions drawn based on partially observed data. For all of these reasons, it is important to think carefully about statistical approaches to interpreting delirium trials. We have communicated previously on this subject [1]; the goal of the present paper is to expand further on statistical topics arising in delirium research.

The approach is chronological, and the typical phases of a delirium trial, from planning to analysis and trial reporting, are discussed in turn. Selected statistical methods are reviewed, along with their underlying assumptions and the implications for delirium research. Where appropriate, ideas are illustrated from a historical randomised trial that compared the effectiveness for delirium control of an active treatment against placebo [2].

Throughout, attention is restricted to *randomised* trials, since the most robust evidence for the treatment of delirium is based on the thirteen randomised trials that have been conducted to date (Table 1). Nevertheless, many of our remarks have relevance outside this specific study design. As the body of evidence for the pharmacological treatment of delirium grows, it is to be hoped that the sophistication of statistical methods used will grow in parallel.

## Methods

### Choice of main outcome

The first decision facing investigators in delirium contexts is the selection of the main outcome of interest. In other words, what measurements (or derived quantities) will be used to assess the effectiveness of an intervention? This is necessarily a balancing act between clinical relevance, ease of data collection and statistical convenience: some outcomes, such as the results of psychiatric interviews, are well-established predictors of longer-term patient prognosis but are costly to collect or difficult to analyse, while others may have only short-term relevance but can be gathered and analysed more simply. Many different endpoints have been used in the delirium literature; we consider here only broad classes of endpoint, divided based on their analytical properties.

End-of-trial outcomes are often employed in delirium research. Use of such measures is usually aimed at comparing medium-term prognosis between different treatment groups. This is clearly important from a patient perspective, and may provide the best available estimate of longer-term health status. However, end-of-trial psychiatric assessments are not always a natural choice. The most obvious reason for this is that, for some patients, end-of-trial psychiatric assessments can be suspect or unavailable: patients may discontinue treatment (treatment dropouts, see [3]), data collection may be discontinued (analysis dropouts, see [3]) and, of course, patients may die. Certain dichotomous end-of-trial outcomes, such as recovery or discharge, can be assigned to the subset of patients who die: clearly, death contraindicates both these positive outcomes. However, other reasons for dropout may be less neatly tied to recovery from delirium. A further, more fundamental, reason that standard end-of-trial outcomes are often inappropriate in delirium trials is that an untreated control group also has the potential to improve and recover. Even with no dropouts, then, an end-of-trial comparison of treatment groups may fail to capture the true effects of treatment, which might instead be seen in the speed or trajectory of recovery, not just a single numeric value at the end of a trial.

Similar endpoints carrying somewhat more information are time-to-resolution and time-to-discharge. Unlike dichotomous endpoints, time-to-event outcomes cannot usually be inferred in a simple manner, so we return later to the special analytical tools that are used to handle this kind of censored data. When treatment group differences are small, time-to-event outcomes are arguably less relevant from a patient perspective than end-of-trial outcomes. However, their importance for healthcare providers is potentially substantial; a small improvement in individual time-to-discharge can rapidly accumulate financial and operational benefits, when considered across an organisation.

There are at least two factors that motivate us to consider main outcomes other than those described above. Firstly, in order to conduct a time-to-resolution analysis, delirium resolution needs to be precisely defined. Devlin et al. [4], for instance, acknowledged that no standard definition of resolution exists, but took the reasonable decision to dichotomise a delirium rating scale. This decision is, however, unavoidably subjective, and analyses that do not require setting arbitrary cutpoints may be preferable. Some of the challenges involved in defining delirium resolution are pre-existing cognitive deficits (particularly in those with dementia), the impact of physical illnesses on cognitive abilities, and the high rate of mortality associated with underlying physical pathology.

Even if cutpoints are defined based on external criteria, such as those for the DRS-R-98 score given in [5], the fact that delirium rating scores can (and do) fluctuate above and below any subjective cutpoint calls into question the usefulness of dichotomised scales. Another possibility, then, is to analyse not just an end-of-trial delirium rating score but the *set* of all scores associated with a patient. Analysis of longitudinal trajectories again requires specialised tools, which we describe later, for understanding the effects of treatment.

### Design

There is another reason to avoid dichotomisation, if possible. Trzepacz et al. argue that difficulty in enrolling patients in delirium trials will mean that sample sizes tend to be small [6]. For this reason it is desirable to maximise the information contribution of every patient. Dichotomised scores clearly provide less detail for analysis than the score itself, and the longitudinal *trajectory* carries more information than either time-to-event or single-outcome data.

Of course, multi-centre trials can overcome some of the difficulties in recruiting sufficient patients to conduct delirium research. This is especially advantageous if a study of subgroups is desired; risk factors vary widely between patients, and stratified randomisation may help to understand the effects of treatment; for instance, when delirium is brought on by pneumonia. Nevertheless, it seems clear to us, even given a large sample, that we should prefer efficient uses of whatever information is gathered. Studies using longitudinal trajectories as the main outcome will almost always have greater power than end-of-trial based analyses.

If longitudinal trajectories are to be the main outcome of interest, then it is worth giving special consideration to the planned observation schedule. Typically, measurements are collected more frequently towards the start of a trial, with lengthening intervals between waves of data collection as the trial progresses. However, our recommendation is to have at least two periods of high-frequency observation; this provides useful information in understanding the variation in trajectories of both early and late dropouts from trials, which often are qualitatively different one from another. Among patients treated in a trial setting, early dropouts are often largely composed of acute cases, while later dropouts are more often due to transfer or recovery.

Design, like the main outcome, cannot usefully be chosen in isolation, and must be conducted with reference to the proposed statistical analysis, of which more discussion later.

### Data collection

When attrition is a real possibility, an important aspect of data collection is that it should not be limited to the main outcome. Whenever all or part of the main outcome data may be missing, it is useful to know as much as possible about the patient before, and after, the missed measurement. This auxiliary information may take the form of simpler indicators of delirium status, prose descriptions of the reason that the patient or clinician decided to terminate treatment or data collection, comorbidities, causes of death, or extra-trial medication. Any or all of these may contribute to a better appraisal of the plausibility, or otherwise, of the competing assumptions underlying the different procedures that can accommodate partial outcome data, to which we now turn.

### Analysis

Although Adamis has outlined several statistical methods that may be useful for the analysis of treatment trials [7], only three recent studies considered trial dropouts in detail as part of the statistical analysis [2,4,8]. In this section, we describe four techniques that, in quite different ways, overcome some of the limitations of data truncated by dropout or death.

As an illustration of the practical application of these statistical approaches, we present results from a trial of an active pharmacological agent for the treatment of delirium. Details on the trial itself can be found elsewhere [2]; briefly, 42 patients were randomised to one of two treatment groups, and the Delirium Rating Scale Revised 98 (DRS-R-98) was used as the primary outcome measure [5]. We focus here on the DRS-R-98 severity scores.

Our selection of four analytical approaches is by no means exhaustive. The statistical literature surrounding generic missing data, and dropout in particular, continues to grow apace [9]. One important consensus among missing data specialists, though, is that it is prudent to consider how results might change if different statistical assumptions were adopted. This *sensitivity analysis* can be conducted formally, by varying the inputs to a statistical model, or informally, simply by employing multiple analytical approaches. We illustrate the latter approach using the historical trial data.

In commending multiple analyses as a means of exploring sensitivity to assumptions about dropouts, we should be clear that we are not proposing a post hoc search for a desirable finding. If more than one analysis is conducted then more than one analysis should be reported. If their results are broadly similar, we gain extra confidence in our reporting; if they differ substantively, then the role of the auxiliary information gathered alongside the main outcome becomes even more important in selecting between competing techniques, if indeed any are appropriate.

Generally, we argue for a flexible approach to the analysis of trial data. While there are evidently some aspects of an analysis plan that can and should be specified pre-trial (the main outcome and proposed subgroup analyses, for instance), there are also components of a statistical model that ought to be inferred from the data. In particular, it is difficult and unnecessary to prespecify the likely form of overall and individual longitudinal trajectories, which can instead be explored by a data scientist with a view to optimising the fit of the model to the data.

#### Time-to-event analysis

Devlin et al. report on a pilot placebo-controlled trial in an intensive care context [4], and use time-to-resolution from delirium as their main outcome. In common with almost all time-to-event analyses, not everyone experiences the event (in this case, resolution), so the approach of Devlin et al. admits study dropouts in a very natural fashion: they are treated as censored observations, individuals for whom we do not know the resolution time. Instead, we know only that their resolution time is greater than the time they were under observation, before dropping out. The central assumption underlying time-to-event analyses is that the dropouts must not be systematically different from those whose delirium is resolved while under observation. This supposition would be violated if (say) healthier individuals discharged themselves before delirium resolution could be conclusively determined. If the assumption of representative censoring is thought to hold, then the partially observed patients may still contribute to the statistical analysis until the point of censoring. Table 1 illustrates that time-to-event analyses are not uncommon in publications concerned with delirium, though the care with which these are undertaken varies from study to study.

To illustrate the approach, we carried out a time-to-resolution analysis of historical trial data [2]. Devlin et al. used a log-rank test based on censored resolution information to compare active treatment with placebo groups; we prefer to use a (so-called) accelerated failure time (AFT) regression model to estimate the difference in rates of recovery between the two groups [10] (p44). Our feeling is that a difference in recovery rates is a useful, interpretable quantity, having relevance for both clinicians and patients.

**Fig. 1:**
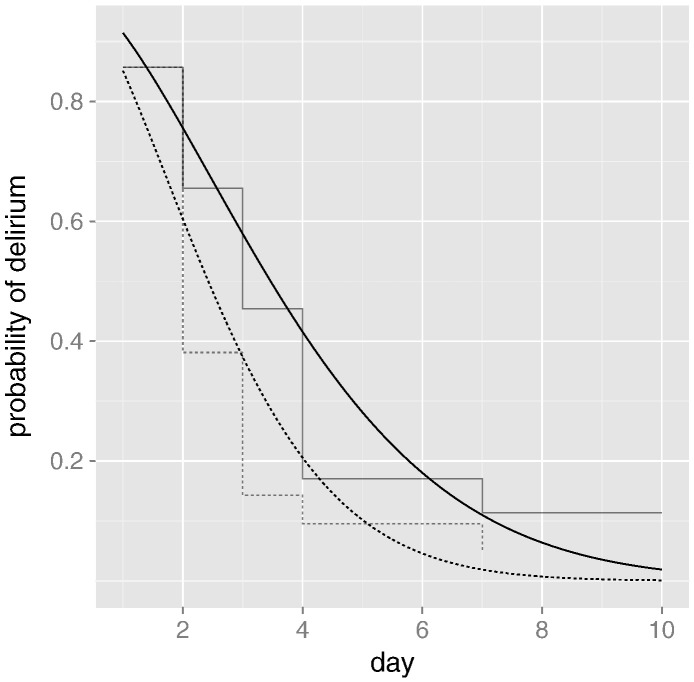
Probability of delirium. The smooth curves are model-based estimates, with the solid line representing the placebo group and the dashed line representing the active treatment group. Lighter shades show their Kaplan–Meier equivalents.

To carry out a time-to-resolution analysis, we must (of course) first define resolution of an episode of delirium. Here we use the severity score of the DRS-R-98, and define resolution as the first measurement less than 13. Using this definition and an AFT model, we estimate that the active treatment group recovered 42.9% more quickly than the placebo group (95% CI − 3.5% to 111.6%, *p*-value .07) over the course of the trial. In other words, the proportion recovered in the active treatment group at day 7 would not be expected to be matched by that in the control group until day 10 (10 ≈ 1.429 × 7). Fig. 1 shows the model-based and non-parametric Kaplan–Meier estimates of the probability of delirium over the first 10 days of the trial.

As we have already said, many other definitions of recovery are possible, including those that combine multiple scales or subscales. We therefore examined several other possibilities, details and specific results which are available from the authors. Unfortunately, the estimated difference in recovery rates is highly sensitive to choice of definition of resolution. A substantial majority of analyses favour the active treatment, some very strongly so, but this is not uniformly true. In our view, this is a disadvantage of the time-to-resolution approach: at the very least, it depends on a subjective decision about what constitutes recovery, and is potentially wasteful of valuable information. In the absence of an established definition of resolution of delirium, there is the possibility of failing to recognise a real improvement in resolution time or, conversely, detecting an apparent, but spurious, effect of treatment. A further disadvantage of this type of analysis is that if, as in this case, DRS-R-98 is only measured relatively infrequently, we may not be able to accurately specify the time of resolution. If, for instance, an individual's severity score is 23 at day 10 but 12 at day 30, we lack a clear idea of when ‘recovery’ took place. This is known generically as interval censoring, and usually adds to the complexity of a time-to-resolution analysis.

#### Explicit imputation

Another approach to missing data problems involves the analyst filling in the unrecorded values, known generically as imputation. Imputation can be a useful procedure, since the plausibility of the imputed trajectories can be explicitly examined, and since standard statistical methods can be brought to bear on the resulting ‘complete’ data. Clearly, the success of such a strategy depends strongly on the skill of the analyst in reconstructing what might otherwise have happened, had the patient not dropped out. By no means is this straightforward, and general prescriptions for meaningful imputations are difficult. Here, we restrict ourselves to a cautionary note about two variants of a commonly-employed imputation strategy, and make two suggestions about how these simple imputations might be improved upon in the delirium context.

As their names suggest, Baseline Observation Carried Forward (BOCF) and Last Observation Carried Forward (LOCF) fill in the missing values with, respectively, the initial or most recently recorded measurement. The obvious advantage of B/LOCF is simplicity of implementation; however, it is important to realise that simplicity of interpretation does not necessarily follow. Criticisms of these imputation mechanisms abound [7,9], but it is particularly easy to see why they will fail to have much relevance in trials of delirium. Recovery is expected in most delirium patients, but these imputations suggest instead that the most recently observed value (or, worse, the initial measurement) will be maintained indefinitely. This is quite implausible, and hence highly likely to introduce bias into any analyses conducted on the basis of the imputed data.

To impute measurements that more adequately reflect a general trend towards improved psychiatric condition, one possibility is to find the midpoint between the most recent patient-specific observation and the mean of all available (genuine, unimputed) observations at the measurement time of interest. This affords the analyst a compromise between acknowledging, on the one hand, that between-patient variability is likely to be high, and on the other that patients tend to follow similar recovery trajectories. We call this approach “realistic mean” imputation, and proceed to contrast it with the BOCF and LOCF imputations in the simple example shown in Table 2. A refinement of this idea would impute a recovery trajectory among those who are analysis dropouts but a worsening trajectory among those who go on to die; we do not pursue this idea further here.

The BOCF imputation at day 4 (20) is manifestly unlikely, while the LOCF imputation (14) is scarcely better. Imputation of the completer mean (8) is another possibility, but fails to recognise that this individual is making a slower-than-average recovery. At least in this instance, the realistic mean imputation (11 = (14 + 8) / 2) seems to us to earn its name: it may not be real, but it is realistic. Further missing values can be imputed sequentially, taking the average of the most recent imputation and the current completer mean. A related idea, called Last Residual Carried Forward (LRCF) finds the most recent subject-specific deviation from the completer mean (2 = 14 − 12) and then adds this to the completer mean at the relevant point in time (10 = 8 + 2) [9].

Two features are common to all of the imputations discussed so far: all are single, deterministic imputations and all are nonparametric (that is, no formal statistical model is used in their computation). Of these, the first is probably the greater weakness, since failure to convey the uncertainty surrounding our imputations in turn risks overstating the precision of our conclusions. Multiple imputation, then, adds a stochastic (that is, random) component to whatever procedure is used to fill in the missing values, and conclusions are averaged across several versions of the in-filled data. There exist simple rules for quantifying the uncertainty inherent in multiply-imputed analyses [11].

**Fig. 2:**
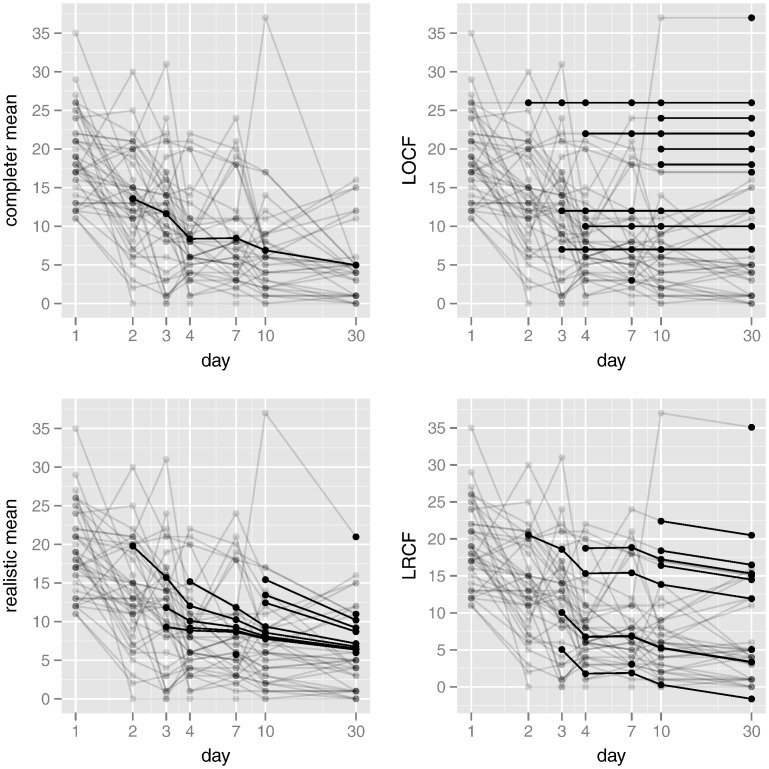
Imputation plots. Imputed trajectories are shown as dark lines, with observed trajectories in a lighter shade.

**Fig. 3:**
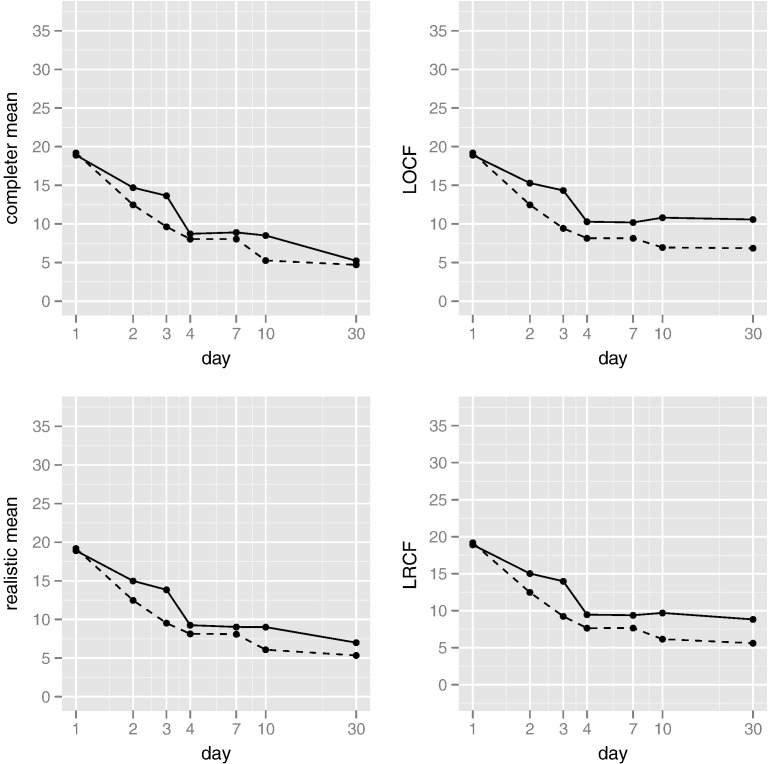
Group mean plots. The placebo group is shown as a solid line, with the active treatment group shown as a dashed line.

In principle, imputation (single or multiple) allows the analyst to fit whatever statistical model they desire, since the data are explicitly completed. We reiterate that the usefulness of these inferences is directly related to the quality of the imputation procedure. We illustrate the foregoing four imputations here, in order to directly examine the plausibility of imputed recovery trajectories. Fig. 2 shows the resulting imputations for all 13 individuals for whom some data were missing. Imputed data are shown in bold, while observed trajectories (for all 42 participants) appear in light grey. Imputation was blinded to treatment, so the imputations that require knowledge of the completer mean – that is, all except LOCF – were based upon averages arising from subjects in both treatment arms. It is clear that imputation of the completer mean fails to adequately capture the variability in the profiles, while LOCF does not reflect the overall trend towards recovery. Both realistic mean and LRCF imputations are more plausible, but it is our belief that the end-of-study stability of the realistic mean imputations is desirable. Since our goal in this section is purely illustrative, we refrain from adding a random component and making multiple imputations. Given these imputations, we can compare the average levels of the DRS-R-98 severity score between the two treatment groups. Irrespective of the imputation used, very little evidence of any end-of-trial treatment differences was uncovered: none of the imputations showed a significant difference between mean levels at day 30. However, as Fig. 3 illustrates, there was some suggestion of a difference in mean levels earlier in the trial. In particular, at day 3 and across all imputations, the mean of the active treatment group was significantly lower than the mean of the placebo group. This consistency is unsurprising, since only three observations were missing at this point in time (see Fig. 2). As Table 3 illustrates, the estimated differences in mean levels at day 3 ranged from − 4.90 (LOCF) to − 4.03 (completer mean), with the realistic mean and LRCT imputations intermediate to these values. Recall from our discussion of multiple imputation that single imputation (as we have done here) is likely to overstate somewhat the precision of our findings; nevertheless, the magnitudes reported in Table 3 would remain unaffected.

In isolation, significant differences at day 3 but nonsignificant differences at day 30 make it tempting to conclude that the effect of the pharmacological agent must therefore be limited to the early portion of the trial, but (as we shall see) other interpretations are possible. A potential disadvantage of realistic mean imputations is that the imputed recovery trajectories will necessarily stabilise at ‘true’ recovery, whatever that may look like; this lack of variation may be unrealistic. A corresponding disadvantage of LRCF imputation is that it is quite possible to impute measurements that do not respect the limits of the scale: negative DRS-R-98 severity scores, for example. Despite the fact that both realistic mean and LRCF are (in this sense) somewhat naïve imputations, they both represent a simple and substantial improvement over the manifestly inappropriate BOCF and LOCF options. More sophisticated options exist (for example, [12]), but typically require consultation with a statistician.

#### Linear models

Because generating believable imputations is a difficult business, some practitioners prefer not to directly impute, but instead rely on more generic assumptions concerning the nature of the mechanism that leads to subjects dropping out of a trial. It is to this kind of approach that we now turn.

One prominent type of statistical inference depends on calculating the values of model parameters that maximise a mathematical function called the likelihood. There exist widely-known conditions on the dropout mechanism under which likelihood-based procedures retain the desirable property that conclusions are unbiased in large samples [13]. Precise specification of these conditions is somewhat technical but, roughly, provided patients drop out (or are removed from a trial by attending physicians) on the basis of observed measurements only, then the dropout is said to be ‘missing at random’ and likelihood inferences are valid. Put more informally, data are missing at random if whatever caused the patient to drop out does not depend on the missing outcome itself. In this sense, missingness at random is similar to the representative censoring assumption used in time-to-event analyses.

The missing at random supposition is always hard to verify empirically, but we comment briefly on its plausibility in our historical data. Of the 42 individuals randomised, 13 dropped out of the study before day 30. Of these 13 dropouts, 4 died; mortality raises challenging philosophical questions regarding the precise scientific objective [14]. One patient left the study before beginning treatment. Missingness due to non-compliance (in this case, 3 patients) or physician intervention (3 patients) can often be assumed to be (approximately) at random, because individual decisions are usually taken on the basis of information available prior to dropping out. The remaining 2 patients experienced adverse events; for them, the assumption of missingness at random is less plausible, since presumably these events may also have been reflected in the (unobserved) DRS-R-98 severity scores. With this qualification, we proceed with the analysis under the assumption of random missingness.

Crucially, if dropout is missing at random, then no formal imputation of missing values is required. However, the statistical model employed must be able to accommodate sequences of observations that vary in length between individuals. One such is the so-called ‘random effects’ or ‘mixed effects’ model, sometimes known more generically as a repeated measures analysis. Random effects models allow for the fact that the measurements making up an individual's longitudinal trajectory are correlated: if a patient scores highly on a delirium rating scale at baseline, they are more likely to continue to score highly throughout. Random effects models are particularly well suited to characterising variation between individuals, allowing some patients to be higher-than-average or faster-than-average responders throughout the course of a trial (and conversely). The simplest form of random effects model is a variant of linear regression; linear mixed effects models offer many advantages, including stable estimation and straightforward relationships between population averages and individual responses. Maximum likelihood techniques can be used to fit mixed effects models, a useful approach provided that dropout can be assumed to be missing at random.

In the historical trial data, we can allow for different mean values for both treatment groups and each observation occasion, and account for correlation within a patient's observations by introducing a random linear trajectory, the variance of which we estimate. The overall mean trajectories are very similar to those shown in Fig. 3. Rather than report individual coefficients, we focus on a global measure of improvement: the average difference between treatment groups, across all measurement occasions. On average, the active treatment group was 2.05 points lower on the DRS-R-98 severity scale (95% CI − 0.37 to 4.47, *p*-value .097), demonstrating weak evidence for an effect on delirium severity across the whole period of the trial. However, since this comparison gives equal weight to every observation point, it will tend to underestimate treatment effects on a self-limiting condition such as delirium.

#### Nonlinear models

Recall that the two main advantages of the time-to-resolution approach to analysis of delirium trials are the ease with which data from subjects who drop out may be included, and the natural way in which the speed of recovery may be compared between the treatment groups. Linear mixed effects models share the first advantage, but do not provide a natural framework for comparing speed of recovery. For this, we must turn to nonlinear models.

Nonlinear models abandon the restrictive assumption that parameters relate in a simple way to the measured responses, at the cost of more arduous estimation and added complexity in mathematical interpretation. However, they offer one enormous advantage: model parameters can be (and usually are) quantities of direct scientific interest, such as a difference in recovery speeds between two treatment groups. Consequently, although more troublesome from a statistical perspective, nonlinear models are often more appealing from a scientific point of view. Whether or not this advantage is sufficient to outweigh the disadvantages inherent to nonlinear modelling is inevitably application-specific.

The historical trial data were gathered with a simplistic cross-sectional main outcome measure in mind. However, since longitudinal data were gathered, we felt strongly that we ought to (a) make use of all available data; (b) avoid subjective dichotomisation; (c) accommodate incomplete data records; and (d) directly compare the speed of recovery of our two treatment groups, leading to our ultimate choice to present both the prespecified analysis *and* to fit nonlinear mixed effects models [2]. Like the time-to-resolution analysis, it aims to estimate the difference in recovery rates between the two treatment groups; like the linear mixed model, it is based on a ‘missing at random’ assumption and does not explicitly impute any data. The model we used incorporated terms quantifying the rate of progression towards recovery in the placebo group, the acceleration in recovery in the active treatment group, a common mean at the start of the trial and a shared long-term prognosis, together with between-patient and within-patient sources of variation.

**Fig. 4:**
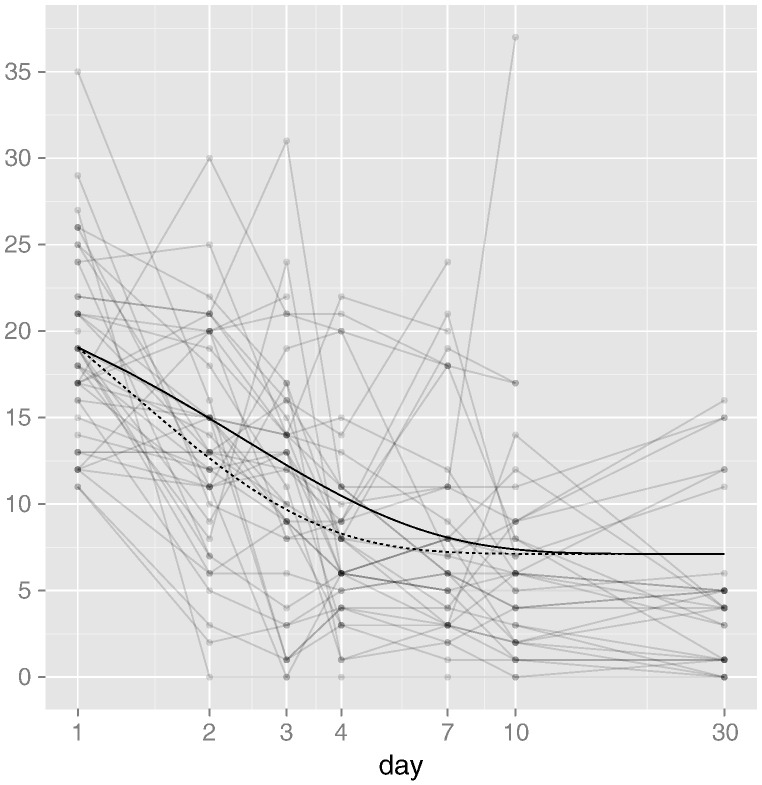
Nonlinear model trajectories. The estimated placebo group mean is shown as a solid line, with the estimated active treatment group mean shown as a dashed line.

An extreme observation in the placebo group at day 10 has a noticeable effect on the rate difference (in favour of the active treatment). Deleting this single observation in order to make our findings more robust, the difference in recovery rate is estimated to be 0.827 (*p*-value .026). This figure (82.7%) is directly comparable to the 42.9% improvement in the time-to-resolution analysis, and in particular the mean level in the active treatment group at day 4 would not be expected to be reached in the placebo group until day 7 (7 ≈ 1.827 × 4). However, the difference is perhaps most easily interpreted with reference to Fig. 4, showing the model-based estimates of the mean recovery trajectories in the two treatment groups.

Notice how the mean trajectories estimated from the nonlinear model capture many of the features discussed throughout this paper: the largest difference at any single point in time is observed around day 3; most of the impact of the active treatment is seen before day 10; by day 30, the mean of both treatment groups has stabilised around 7 points on the DRS-R-98 severity scale. Both between and within patients, there is substantial variation around these mean trajectories, and no data have been imputed. Nonetheless, by employing a statistical model that is well-suited to the features of delirium trials we have discussed, we are able to better explore the clinical implications of introducing the pharmacological agent on the *rate* of recovery on the DRS-R-98 severity scale.

### Sensitivity analysis

To our knowledge, this is the only comparative analysis of trial data in a delirium setting. Different statistical approaches highlight different aspects of the data: imputation averages and linear models emphasise differences in group means, while the time-to-event and nonlinear models focus on progression towards recovery. Reassuringly, there is a large degree of consistency between the analyses reported here. Taken together, the quantitative results seem to us to paint a convincing qualitative picture that – at least in this instance – the active treatment has had a beneficial effect on the treated group.

## Discussion

In this paper, we have considered several statistical points of view on the same scientific problem: estimating the effect of treatment in delirium trials, especially when some observations may be missing. As will be evident from the foregoing illustrative analyses, our recommendations are based not only on successes, but also on the mistakes that we have made, and learned from, in our own trial work.

We have chosen to illustrate the statistical methods that we consider to be most applicable to delirium trials. There are, of course, many other possibilities, ranging from slight variations on these themes to entirely different perspectives. More broadly, then, our view is that common features of delirium studies, such as small sample sizes, sample attrition and spontaneous recovery mean that techniques are likely to be preferred if they make efficient use of available data and, more specifically, take a *global* (as opposed to end-of-trial) perspective. This neatly summarises our recommendations to those carrying out delirium research: design, recording and analysis of trials should maintain a global view of the progression of each patient, from the point of recruitment to their ultimate death or discharge and, hopefully, recovery.

Neither have we considered in detail all the statistical issues that arise in delirium trials. For instance, a typical efficacy trial might consider several possible outcome measures, and questions of multiple testing then surface. Also, all the approaches we have discussed rely on an assumption of random missingness [13] or similar; this is a limitation, and remains an active area of statistical research. Much generic advice is available, but we also counsel early consultation with a professional statistician, who can advise on the specific problems that arise in delirium trials, and offer direction on selection and use of an appropriate model.

Given that the body of reliable empirical evidence concerning the treatment of delirium is sparse, it is unfortunate that detailed consideration of analytical aspects of delirium trials is also rare. Ultimately, it can be hoped that sufficient data will emerge to swamp the effects of less suitable mathematical models, but in the present state of fairly limited knowledge (particularly about longer-term outcomes), convincing analyses will be needed in order to influence professional practice. It is our hope that the methods we have described will play a part in convincing analyses of future delirium research.

## Contributions

TT and JB formulated and carried out the historical randomised controlled trial. TT surveyed the literature and proposed and wrote the first draft of the current paper. DF assisted with the analysis of the trial, and described and carried out the statistical methods illustrated in the current paper.

## Conflict of interest

Tahir et al. was an investigator initiated study [2]. In terms of the Clinical Trials Directive, AstraZeneca UK legally sponsored and provided funding for recruitment of a research assistant and for trial medication.

## Figures and Tables

**Table 1:** Randomised trials for treatment of delirium. Principal statistical approaches used by the authors are also indicated. *n* = sample size, *d* = dropouts

Study	Drug	*n*	*d*	Population	Methods
Breitbart et al. (1996) [15]	Haloperidol vs.	11	2	AIDS	ANOVA
	Chlorpromazine vs.	13	2		
	Lorazepam	6	6		
Han and Kim (2004) [16]	Haloperidol vs.	14	2	Mixed medical–surgical	ANOVA
	Risperidone	14	2		
Skrobik et al. (2004) [17]	Haloperidol vs.	45	?	Medical and surgical ITU	*t*-Test, ANOVA
	Olanzapine	28	?		
Kim et al. (2005) [18]	Haloperidol vs.	24	?	Medical, oncology ITU	Time-to-event
	Risperidone	18	?		
Lee et al. (2005) [19]	Amisulpride vs.	20	4	Medical, ITU and oncology	Time-to-event
	Quetiapine	20	5		
Hu et al. (2006) [20]	Olanzapine vs.	74	1	Senile dementia	*t*-Test
	Haloperidol vs.	72	4		
	Control	29	18		
Devlin et al. (2010) [4]	Quetiapine vs.	18	0	Intensive care unit	Time-to-event
	Placebo	18	2		
Tahir et al. (2010) [2]	Quetiapine vs.	21	5	General hospital	Nonlinear model
	Placebo	21	8		
Girard et al. (2010) [21]	Haloperidol vs.	35	2	Intensive care unit	Time-to-event, GEE
	Ziprasidone vs.	32	2		
	placebo	36	2		
Kim et al. (2010) [22]	Risperidone vs.	17	5	General hospital	LOCF, time-to-event
	Olanzapine	15	7		
van Eijk et al. (2010) [8]	Rivastigmine vs.	55	19	Intensive care unit	Time-to-event
	Placebo	54	15		
Overshott et al. (2010) [23]	Rivastigmine vs.	8	1	Medical wards	*t*-Test
	Placebo	7	4		
Grover et al. (2011) [24]	Haloperidol vs.	26	6	General hospital	ANOVA
	Olanzapine vs.	26	3		
	Risperidone	22	1		

**Table 2:** Example individual and average trajectories for DRS-R-98 severity score

	Day 1	Day 2	Day 3	Day 4
Patient raw data	20	17	14	?
Completer mean	22	16	12	8

**Table 3:** Imputed mean trajectories. Within each imputation type, the mean of the placebo group (Plac.) is shown on the left, with the mean of the active treatment group (Act.) on the right

Day	Observed mean		LOCF		Realistic mean		LRCF	
	Plac.	Act.	Plac.	Act.	Plac.	Act.	Plac.	Act.
1	18.905	19.190	18.905	19.190	18.905	19.190	18.905	19.190
2	14.695	12.476	15.286	12.476	14.990	12.476	15.026	12.476
3	13.650	9.632	14.333	9.429	13.844	9.531	13.981	9.243
4	8.721	8.036	10.286	8.143	9.258	8.140	9.467	7.647
7	8.899	8.045	10.190	8.143	9.031	8.093	9.390	7.656
10	8.501	5.263	10.810	6.952	9.011	6.083	9.701	6.163
30	5.227	4.706	10.571	6.857	7.000	5.347	8.825	5.611
